# Pedagogy of Success: Perception in undergraduate and postgraduate students at a Peruvian university

**DOI:** 10.12688/f1000research.55310.1

**Published:** 2021-11-16

**Authors:** Silvia Ana Valverde-Zavaleta, Ray Harvey Mellin Rubio, Aurea Elizabeth Rafael Sánchez, Martha Gonzales Loli, Enaidy Reynosa Navarro

**Affiliations:** 1 Universidad Católica de Trujillo Benedicto XVI, Trujillo, 13001, Peru; 2 Universidad Nacional de Trujillo, Trujillo, 13001, Peru; 3 Universidad Nacional Federico Villareal, Lima, 15088, Peru; 4 Universidad César Vallejo, Trujillo, 13001, Peru

**Keywords:** Pedagogy; Success; Perception; Undergraduate; Postgraduate; Affectivity

## Abstract

Background: The study's objective was to compare whether there are differences in the perception of undergraduate and postgraduate students about the pedagogy of success.

Methods: This is non-experimental research with a comparative descriptive design, and a hypothetical deductive method was used. The perception of success pedagogy questionnaire was applied as a data collection technique with a sample of 50 university students with 23 items based on three dimensions: opportunity to learn (nine items), feedback (eight items), and consideration of the person (six items), with the following qualitative value scale, always = high, sometimes = middle, and never = low; the scale of quantitative value was from two to zero. To find the instrument's validity, it was subjected to expert judgment, calculating the content validity ratio for each item and considering the criteria of clarity and relevance. The instrument's reliability was determined using Cronbach's alpha, which found a high consistency between the items. For the questionnaire application, Google Forms was used to obtain fast, timely, and reliable answers. The data were processed through the SPSS V. 25.

Results: The pedagogical support of the postgraduate teacher is more effective than that of the undergraduate. The undergraduate teacher stands out for listening and being more empathic. Didactics is crucial for students to develop their cognitive and human potential. Academic success is related to the teacher's pedagogical skills and the student's motivation. In addition, teachers and students can develop cognitive skills through effective communication and socialization. Finally, the affective dimension allows students to achieve personal and professional goals.

Conclusions: There are significant differences in the perception of the pedagogy of success among undergraduate and postgraduate university students; therefore, the application of this methodology is more promoted towards postgraduate students than to their undergraduate peers.

## Introduction

An essential part of the purpose of university education is ensuring that students can know how to be, know how to know, and learn how to do. ^1^ Teachers must generate improved didactics in their pedagogical work, ^2^ allowing students to develop these educational competencies. ^3^

Theoretically, this study is based on Juan Amos Comenius’ research on pedagogy. Comenius argued that pedagogy is a science with a strict educational purpose that integrates educational materials to achieve quality teaching that impacts the person and the surrounding society. He also argued that academic success was guaranteed from horizontal and effective communication with his students ^4^, allowing them to develop their potential cognitive abilities, interrelate, collaborate and interconnect. ^5^

The pedagogy of success is based on didactic strategies that motivate the learner to manage their learning, promoting; respect for diversity, empathy, harmonious coexistence, and the use of ICT ^6^ to avoid failure or the feeling of academic loss. ^7^ For this, teachers trained in emotional intelligence issues (motivation, self-regulation, self-knowledge, empathy, and social skills) are needed. ^8^ In conclusion, educators must be capable of forming tolerant, respectful, democratic, caring, and competent citizens that can perform professionally in a complex and changing world. ^9^

In the pedagogical task, educators focus on achieving two basic pedagogical principles. ^10^ The first principle is based on providing a supportive environment where the learners can get involved; the second is based on developing practical classroom activities. In this regard, the following aspects are systematized: a) Errors resulting from experimentation are a part of learning. Therefore, the teacher must assist the student in overcoming mistakes. b) Outstanding achievements are reinforced to improve the student's self-esteem and motivate them to continue learning. c) Importance should be given to general activities without preventing the learner from excelling in specific activities. d) Self-correction helps the learner to be critical of their work and to be independent. e) The teacher must address competitiveness as a central point of the training process and generate a collaborative work environment where they share their successes. f) Parents, teachers, tutors, classmates, and socio-cultural space, intervene in the formative process of the student. g) The educational content that the teacher develops with their students must be attractive and, at the same time, reflect the socio-cultural reality of the student. h) Students actively participate in the learning process to achieve their goals. i) Classroom activities must involve everyone without neglecting the rhythm and learning style of each person. j) Work must be evaluated on an individual basis, and evaluation techniques must be adapted with the learning objectives.

Learning allows students to discover their passions and goals. The teacher accompanies the students in this process of discovery and encourages them to build their future using the knowledge gained and applying it to current societal problems. Thus, successful teaching is correlated with the quality of teacher training. ^11^ Teaching is a real challenge, especially when teachers do not have the required skills to implement change. ^12^

## Literature review

Rompelmann (2002) proposes three components to strengthen the pedagogy of success: 1) opportunity for response, 2) feedback, and 3) consideration for the person. ^13^ The first component considers equity in the opportunity to respond, individual support for the student, latency, deepening, and high expectations in reasoning. The second involves evaluating, correcting, praising, providing reasons for the recognition, and active listening. The latter includes proximity, courtesy, respect, a caring approach, and limits to behavior. The correlational and multiple regression analysis developed by Ali Roohani and Shahrzad Haghparast (2020) showed a significant and positive relationship between critical pedagogy and teachers' reflective thinking. Both variables were predictors of pedagogical success from the evaluation of the students. ^11^ The research by Galbán Lozano and Ortega Barba (2021) highlights that a fundamental quality of the teacher is the vocation for teaching and the confidence and concern that they demonstrate for the student's academic development. The study also points out that students feel satisfied and identify better with those teachers who, without neglecting the theoretical-practical link, promote environments for active learning. ^14^

University teachers, above all, must be specialists in their subject area. They must also be motivated to develop teaching and scientific research with enthusiasm, interest, and vocation and show tolerance, empathy, justice, openness, creativity, flexibility, availability, and curiosity in otherness. They must possess social skills for effective communication and management of difficult emotions. Organization and effective structuring of knowledge is also crucial, along with short and long-term planning and promoting independent and critical thinking. They should demonstrate an acute and reflective attitude about their performance and be innovative and adaptable. ^15^

The student’s journey is characterized by their cognitive and motivational development and the challenges of university life. Faced with this, the educator needs the pedagogical tools to accompany them in complex issues such as lack of motivation, communication problems, stress management, teamwork deficiencies, and accepting change. The student needs an approach to the modern educational reality to understand, reflect and merge the acquired knowledge, forming values that promote solidarity, friendship, respect, cooperation, and joint creation as fundamental learning.

The present investigation is justified from a set of theoretical antecedents and epistemological arguments that allow us to understand undergraduate and postgraduate students' perceptions of the teacher's pedagogical development. It presents a design that will enable the results to be interpreted, statistically processed, and replicated in other educational contexts. From the practical point of view, it provides new knowledge and approaches that help the comprehensive professional training of undergraduate and postgraduate students, regardless of their perception. Likewise, it offers pedagogical and personal development reflections relevant to minimizing the difficulties that affect students' desirable academic skills: knowing, doing, and acting, allowing them to align their potential intellectual development with aspirations for success. Overall, this research highlights the relevance of the pedagogy of success in the comprehensive training of teachers and students.

The study's objective was to compare whether there are differences in the perception of undergraduate and postgraduate university students about the pedagogy of success. The general hypothesis holds that there are differences in the perception of success among undergraduate and postgraduate university students.

## Methods

### Study design

This study was non-experimental with a comparative descriptive design. The hypothetical-deductive method was used, whereby the variable's dimensions are known through observation, deduction, verification, or factual verification of the facts. ^16^ The pedagogy of success variable was structured by three dimensions: opportunity to learn, feedback, and consideration of the person. Participants were undergraduate and postgraduate students enrolled at the Universidad Católica de Trujillo, Peru (UCT, Peru).

### Study population

Students were approached to take part in the study via institutional email and social networks. Those who agreed to participate were then invited to a meeting held via Zoom, in which they were formally invited to the study. All participants were informed about the importance of the study, the anonymous nature of the instrument, and the theoretical, methodological, and practical procedures that would be used to answer the research question. It was also explained that the results of the said study would serve as the basis for developing quality education focused on improving the perception of success of undergraduate and graduate university students at UCT Peru. The population was 50 students: 20 undergraduates, X cycle (fifth year, professional career of Initial Education, Faculty of Humanities, UCT, Peru), and 30 postgraduates, IV cycle (second year, Master of Education Postgraduate School, UCT, Peru); total sample: 100% of the population. The inclusion criteria for participants were: Undergraduate students pursuing the professional career of Initial Education and postgraduate students pursuing a Master of Education. All participants had to be enrolled at UCT, Peru. In the case of undergraduate students, they had to be studying the tenth cycle of studies, and postgraduate students, the second cycle. The sample was non-probabilistic for convenience; it was selected in this way, taking into account restrictions on in-person meetings in the COVID-19 context. All participants were enrolled at UCT, Peru, located on North Panamericana Avenue. 555, Moche 13600, Trujillo, La Libertad, Peru.

We successfully used 100% of the sample data after recruiting all participants in a short space of time and processing the data immediately. All participants gave their informed and signed consent as an essential requirement to participate in the study.

### Data collection

The data collection technique was a questionnaire. ^22^ As the study instrument, the Perception of Success Pedagogy questionnaire was administered, which consisted of 23 items distributed in three dimensions: opportunity to learn (nine items), feedback (eight items), and consideration of the person (six items). The qualitative value scale used was: high, middle, and low; likewise, the quantitative value scale was two to zero. To test the general hypothesis, the nonparametric Mann-Whitney U test was used. Due to social distancing, the decision was made to distribute the questionnaire through Google Forms. The reasons for this decision were communicated to the participants through a meeting held on February 25, 2021, via Zoom. This meeting was called and directed by the team of researchers. Subsequently, the questionnaire was sent to all participants through their institutional emails so that they could respond to them within approximately 20 days. The data collection period began March 1, 2021, and ended April 22, 2021.

The data sources were: 1). Enrollment records of undergraduate and postgraduate students, UCT, Peru; cycles X (undergraduate) and IV (graduate). 2). Results of the applied questionnaire. To avoid potential biases, the instrument was validated. First, pilot testing was carried out, then it was submitted for expert assessment. The instrument's reliability was performed using the Cronbach's Alpha test, obtaining (0.887). The pilot test was subjected to expert judgment to calculate the content validity for each item, considering the criteria of clarity, belonging, and relevance. The experts concluded that the test was valid. Therefore, they recommended not to delete any item and apply it. To compare the pedagogy of success between the undergraduate and postgraduate groups, the parametric student's t-test was used for independent samples.

### Analysis

All data were processed using SPSS V.26 statistical software. The specific processes were carried out as follows: in the Validity and Reliability tests of the instrument, the calculation of the content validity ratio was with the maximum evaluation score of 1 for each item, which was subjected to the validity of V for Aitken. The reliability of the instrument was performed using the Cronbach's Alpha test, obtaining (0.887). To determine normality, the Shapiro-Wilk test was used in the Pedagogy of success variable with p> 0.05; therefore, the null hypothesis was accepted, and the alternative hypothesis was rejected. After contrasting the specific hypotheses in each of them, the results are less than 0.05 with a confidence level of 95%. Regarding the specific hypothesis, for each one, the Mann-Whitney U test was used, the result of which was less than 0.05 with a confidence level of 95%. A copy of the accompanying dataset can be found here. ^22^

### Ethical considerations

The research assumed two fundamental ethical principles: 1. Informed consent, 2. confidentiality and anonymity. ^17^ Before signing the consent letter, the participants received a talk via Zoom, where the study's objective, importance, and scope were explained to them. Participants were informed that the research results would be used strictly for research purposes to improve pedagogical processes. The consent letter was shared with participants via Google Drive and could only be accessed with an institutional email address. This research complies with the Ethical Principles of the teaching staff, provided in Article 6 of the Code of Ethics, Version 1.0; Approved by University Council Resolution No. 143-2019/UCT-CU, dated December 30, 2019, Catholic University of Trujillo, Peru. ^18^

## Results

In Table 1, it can be observed that there are significant differences at p < 0.005 when applying the nonparametric Mann-Whitney U test to the items related to the opportunity to learn dimension.

**Table 1.** Opportunity to learn among undergraduate and postgraduate students.

Dimension	Items	Mann–Whitney U test	Sig.
**Opportunity to learn**	Answer a question, correct or affirm something that has been said in class	125.000	0.000
	Request individual support while working in a group in the classroom	100.000	0.000
	Have individual support outside the classroom or in recesses	115.000	0.000
	Respond in time to a question or question	95.000	0.000
	Have clues or bibliographic references to solve a designated work	130.000	0.000
	Reflect on the proposed topic	90.000	0.000
	Express yourself freely	145.000	0.000
	Raise your own opinions	130.000	0.000
	Present information organizers and explain your productions	155.000	0.001

### Mann–Whitney U test = 69.000, z = −4.685, p = 0.000 (<0.05)

In the comparison of frequencies of the levels of perception of pedagogy of success in the dimension of opportunity to learn among undergraduate and postgraduate students, it is observed that most (65%) undergraduate university students score in the medium level, with the other 35% scoring in the high level. Among postgraduate students, a higher percentage of 86.7% score in the high level for this dimension and 13.3% in the intermediate level. According to the Mann-Whitney U test results, z = −4.685 and p = 0.000 (<0.05), the alternative hypothesis is accepted, concluding that there are significant differences. This suggests that postgraduate students are given more opportunities to learn from the teacher than undergraduate students.

In Table 3, it can be observed that there are significant differences (p <0.05) when applying the nonparametric Mann-Whitney U test to the items related to the feedback dimension.

### Mann–Whitney U test = 99.500, z = −4.009, p = 0.000 (<0.05)

In the comparison of frequencies of the levels of perception of pedagogy of success in the feedback dimension among undergraduate and postgraduate students, it is observed that most (85%) undergraduate university students score in the high level and 15% score in the intermediate level, while among postgraduate students a higher percentage (53.3%) score in the middle level for this dimension, and 46.7% score in the high level. According to the Mann-Whitney U test results, z = −4.009 and p = 0.000 (<0.05), the alternative hypothesis is accepted, concluding that there are significant differences. This suggests that undergraduate teachers provide better feedback on students’ learning compared to postgraduate teachers.

It can be observed that there are significant differences (p < 0.05) when applying the nonparametric Mann-Whitney U test to the items related to the consideration for people dimension.

### Mann–Whitney U test = 169.000, z = −2.634, p = 0.008 (<0.05)

In the comparison of frequencies of the levels of perception of the pedagogy of success in the consideration towards people dimension among undergraduate and postgraduate students, it is observed that most (55%) undergraduate university students score in the high level, and 45% score in the intermediate level, while among postgraduate students a higher percentage of 93.3% score in the high level for this dimension, and 6.7% score in the intermediate level. According to the Mann-Whitney U test results, z = −2.634 and p = 0.008 (<0.05), the alternative hypothesis is accepted, concluding that there are significant differences. This suggests that postgraduate teachers have greater consideration for students than undergraduate teachers.

### Student's t test for independent samples t = −3.270, p = 0.02 (<0.05)

In comparing frequencies of the levels of perception of pedagogy of success among undergraduate and postgraduate students, it is observed that the most significant proportion (50%) of undergraduate university students score in the high level and 20% score in the medium level. In comparison, 96.7% score in the high level for this dimension among postgraduate students and 3.3% score in the medium level. According to the results of the students’ t-test, −3.270 and p = 0.002 (<0.05), the alternative hypothesis is accepted, concluding that there are significant differences. This suggests that the pedagogy of success is promoted more in postgraduate students than in undergraduates.

## Discussion

Higher education teachers must guide their pedagogical performance to help students achieve the general and specific competencies needed for their professional careers. However, to achieve these competencies with undergraduate or postgraduate students, educators must demonstrate didactic qualities, motivate and integrate the student; likewise, they must establish a teacher-student relationship that encourages lifelong learning.

Table 1 indicates that the comparison of frequencies of the levels of perception of the pedagogy of success for the opportunity to learn dimension, there are significant differences (p = 0.000, <0.05); for undergraduate students (65%) with a medium level, and postgraduate students (86.7%) qualify as high level. Galbán Lozano supports these data, and Ortega Barba stated that teachers are professionals who establish connections with students to provide essential support for academic achievement. Only through this method are adequate conditions and academic results created with high well-being and personal satisfaction levels. ^13^ Active listening, learning from mistakes, and evaluating their successes are also emphasized. Similarly, Roohani and Haghparast argued that the quality of teacher training is related to successful teaching. ^11^ These results are theoretically corroborated in previous research that argues that teachers must promote change in their pedagogical practice to create interest in learning. ^2^ It can be seen that academic success is related to the teacher's pedagogical skills and the student's motivation to assimilate new knowledge. ^19^

According to Table 2, the results suggest that in comparing frequencies of the levels of perception of success pedagogy for the feedback dimension, there are highly significant differences (p = 0.000, <0.05). 85% of undergraduate students scored in the high level for feedback, while 53.3% of postgraduate university students scored in the medium level. The teacher must develop didactic strategies that motivate the student in their learning. ^14^ This corresponds with a recent study that considered the teacher's didactic capacity essential to the teacher profile. It emphasizes that university higher education students will succeed if they apply a reinforcing, communicative pedagogy that addresses their doubts and expectations for learning. ^20^ The results are theoretically corroborated. The techniques for achieving pedagogical objectives should consider the student's learning rhythm, the reinforcement of their achievements to improve their self-esteem, and encouragement for the continuity of their studies, emphasizing the students' work. Students value these techniques, and they increase their enthusiasm for other activities. ^9^

**Table 2.** Percentage frequency distribution of perception of success pedagogy concerning the opportunity to learn among participating undergraduate and postgraduate university students.

Pedagogy of success level (opportunity to learn)	Undergraduate students		Postgraduate students	
	N	%	N	%
Low	0	0	0	0
Middle	13	65	4	13.3
High	7	35	26	86.7
Total	20	100	30	100

Table 3 indicates that, in comparing frequencies of the levels of perception of the success pedagogy for the consideration towards people dimension, the data present highly significant differences (p = 0.008, <0.05); 55% of undergraduate students scored in the high level for consideration for people, and 93.3% of postgraduate college students scored in the high level. The findings confirm that teachers and students have the opportunity to develop practical skills through socialization and communication. ^21^ This result is mainly due to the attitude that the teacher has towards the student. If this relationship is appropriate, adequate, and optimal, the probability of achieving affective learning is high. It is also vital to highlight the communication strategies that the teacher applies; the teacher must be passionate about their pedagogical activity. It is not only the application of techniques and instruments, the student must also be considered. The affective dimension is essential for learning because the teacher supports the learner, fosters confidence, professional progress, and enables them to achieve important goals. ^13^

**Table 3.** Feedback received by undergraduate and postgraduate students.

Dimension	Items	Mann–Whitney U test	Sig.
**Feedback**	Positively reinforce your performance	190.000	0.010
	Evaluate your work, highlighting your skills	190.000	0.012
	Congratulate your achievements in the presence of others	165.000	0.001
	Express constructive criticism when presenting your work	200.000	0.022
	When praising your progress, they explain the reasons	165.000	0.002
	During discussions, they invite you to keep talking	145.000	0.000
	Listens to you carefully	205.000	0.024
	Respects your opinions or ideas	125.000	0.000

The results in Table 4 show that in comparing frequencies of the levels of perception of success pedagogy, the data present highly significant differences (p = 0.02, <0.05). 80% of undergraduate students scored high for applying the pedagogy of success, whereas 96.7% of postgraduate university students scored high. These results are also found in a recent study where it was suggested that teachers require personal qualities and skills as university professionals. ^14^ The teacher who pushes for success has a high command of their specialty, is competent in the use of methodological strategies to engage the student and commit them to their learning, has a sense of connection to the student that allows them to mobilize, motivate, and awaken their interest in knowledge and success. All this demonstrates trust, respect, constructive criticism, and support for the optimal learning environment. It should be noted that the pedagogy of success is aimed at avoiding all kinds of educational failure or feelings of academic frustration. It is not necessarily about applying didactic techniques and strategies but also the teacher's way of managing students of varying levels. It is, therefore, necessary for teachers to develop empathy towards students, that is, develop the ability to understand their difficulties in the learning process and give the student the confidence required to overcome adversity. ^9^

**Table 4.** Percentage frequency distribution according to the perception of success pedagogy for feedback among undergraduate and postgraduate university students.

Pedagogy of success level (feedback)	Undergraduate students		Postgraduate students	
	N	%	N	%
Low	0	0	0	0
Middle	3	15	16	53.3
High	17	85	14	46.7
Total	20	100	30	100

**Table 5.** Communication style and empathy towards undergraduate and postgraduate students.

Dimension	Items	Mann–Whitney U test	Sig.
**Consideration towards people**	The teacher's closeness to you	210.000	0.039
	Respect for you	215.000	0.052
	The trust they have with you	255.000	0.302
	Their kindness to you	240.000	0.170
	Affection and consideration for students in class	235.000	0.137
	Demands an academic level without hurting or embarrassing you	210.000	0.039

**Table 6.** Percentage frequency distribution according to the perception of success pedagogy for consideration for people among participating undergraduate and postgraduate university students.

Pedagogy of success level (consideration towards people)	Undergraduate students		Postgraduate students	
	N	%	N	%
Low	0	0	0	0
Middle	9	45	2	6.7
High	11	55	28	93.3
Total	20	100	30	100

**Table 7.** Percentage frequency according to the perception of undergraduate and postgraduate students (general distribution).

Pedagogy of success level (general table)	Undergraduate students		Postgraduate students	
	N	%	N	%
Low	0	0	0	0
Middle	4	20	1	3.3
High	16	80	29	96.7
Total	20	100	30	100

In sum, this research considers an education focused on developing the learner's attitude, motivation, and autonomy, improving the current pedagogy perception in undergraduate and postgraduate students. It provides a relevant theoretical contribution for future research that addresses this issue.

## Conclusions

Once the statistical analysis was carried out, it was determined that there are significant differences in the levels of the Pedagogy of Success for the dimension Opportunity to learn among undergraduate and postgraduate university students; therefore, the postgraduate teacher provides the student with more excellent pedagogical support to improve her learning than the undergraduate teacher. When performing the comparative analysis of the perception of success of Pedagogy in the Feedback dimension, significant differences were found between undergraduate and postgraduate university students; therefore, the undergraduate teacher clarifies, broadens, strengthens knowledge, and gives students more extraordinary listening skills than the postgraduate teacher. In the dimension of Consideration towards people in the perception of successful Pedagogy, significant differences were found between undergraduate and postgraduate university students. Postgraduate teachers were confirmed to provide students with more excellent courtesy, respect, and affection than undergraduate teachers. Finally, it has been shown that there are significant differences in the perception of pedagogical success between undergraduate and postgraduate university students; therefore, in the application of the pedagogy of success as a formal learning process, teaching and personal inspiration of achievements in knowledge management are promoted more to postgraduate students than to undergraduates.

## Data availability

### Underlying data

Zenodo: Pedagogy of success: Perception in undergraduate and postgraduate students at a Peruvian university. https://doi.org/10.5281/zenodo.5307057. ^22^

This project contains the following underlying data:

‐ Statistical Results.xlsx
‐ Processed data.pdf

### Extended data

This project contains the following extended data:

- Pedagogy of success questionnaire.docx
- Success pedagogy questionnaire (Technical sheet).docx

Data are available under the terms of the Creative Commons Attribution 4.0 International license (CC-BY 4.0).
