# Supplementary material for: Pedagogy of Success: Perception in undergraduate and postgraduate students at a Peruvian university
Source: F1000Res. 2021 Nov 16;10:1160. [Version 1] doi: 10.12688/f1000research.55310.1 (PMC8729024; doi:10.12688/f1000research.55310.1)
Supplement: Peer review history

# Peer review: Reviewer response for version 1

**DOI:** 10.5256/f1000research.58874.r100438

**Published:** 2022-01-10

**Authors:** Iris Agustina Jiménez Pitre

**Affiliations:** 1 Universidad de la Guajira, La Guajira, Colombia

This study, on the pedagogy of success and the perception of the participating students regarding this pedagogy; is scientifically sound, it is supported by well-established theoretical and methodological arguments and procedures. The paradigm, approach, and design it presents allow the research to be statistically processed, interpreted and replicated in other educational contexts. The data of this investigation were opportunely shared in open access ( https://doi.org/10.5281/zenodo.5307057).

 One of the main strengths of the study is that it raises an appropriate academic debate to address the significant differences found in the perception of the pedagogy of success among undergraduate and graduate university students. In this sense, delving into this problem will allow students to align their potential intellectual development with their aspirations for success, starting from fundamental pillars such as knowing how to be, knowing how to know, and knowing how to do.

 It is recommended to strengthen the Conclusions of the research because, although they are concise, clear, orderly, and carry an academic language according to scientific requirements; they do not indicate what were the main limitations that were found throughout the investigative process.

Is the work clearly and accurately presented and does it cite the current literature?

Yes

If applicable, is the statistical analysis and its interpretation appropriate?

Yes

Are all the source data underlying the results available to ensure full reproducibility?

Yes

Is the study design appropriate and is the work technically sound?

Yes

Are the conclusions drawn adequately supported by the results?

Partly

Are sufficient details of methods and analysis provided to allow replication by others?

Yes

Reviewer Expertise:

Science and Technology, Science and Technology Project Management, didactics and educational pedagogy.

I confirm that I have read this submission and believe that I have an appropriate level of expertise to confirm that it is of an acceptable scientific standard.

# Peer review: Reviewer response for version 1

**DOI:** 10.5256/f1000research.58874.r100440

**Published:** 2021-12-23

**Authors:** Enrique Renteria

**Affiliations:** 1 Institute of Evaluation and Educational Development, Club UNESCO Compse, Tlalnepantla De Baz, Mexico

The article defines the pedagogy of success with the following characteristics: didactic strategies that motivate the learner to manage his learning; ****promoting respect for diversity, empathy, and harmonious coexistence; and the use of Information and Communication Technologies (TIC). The instrument used confirms that the work achieved by teachers is not done in isolation, but through collegiate work that allows to define a socialized joint work. The questionnaire applied shows that university students perceive this joint work that is defined as teaching. I agree with the conclusion of the objective proposed by the authors in their research, and which is to highlight the relevance of the pedagogy of success in the integral training of teachers and students.

 Teaching as a professional activity has been transformed, like all social practices, significantly with the impact of the conditions of the development of computer and communication technologies, as detailed in my article, "Renteria Castro, Enrique. (2012) *Distance education, the use of new technologies and didactics of university teaching.* Ibero-American Journal of Educational Research and Development". In addition it has moved from a fluctuating dynamic between an empirical and isolated practice, which is perfected with experience, to be considered as a practice intimately articulated to intellectual processes, supported by the previous training and pedagogical didactic training and mainly with the socialization of the analysis of the teaching exercise.

 University teaching becomes a practice based on the reflective and proactive planning of teaching to achieve the design and implementation of strategies for learning, an activity that must be carried out by all teachers, this work of reflection and proposals in the group of teachers is conceptually defined as teaching; and its main action is to be the articulator between theory and social praxis, based on a solid vision of joint work, articulated by two axes; evaluation and processes of continuous improvements.

 This means, according to the authors, that the undergraduate teacher clarifies, broadens, strengthens knowledge and gives students way more extraordinary listening skills than the graduate teacher. With the above, we can lead to a discussion and conclusion that the pedagogy of success consists of achieving with the students the fifth pillar of knowledge of education; learning to transform (UNESCO, 2021, Dialogues on the Futures of Education). Learning that is achieved throughout the formative trajectory of the students and that the motivational level of the graduate students must be considered because of the pedagogical didactic intervention implemented during the undergraduate school cycles.

 It is suggested to consider in the text of Table 4 to refer to the result that consists of the distribution of percentage frequencies according to the perception of success in pedagogy that corresponds to the factor called feedback among university students, which undergraduate students obtain a high score with respect to graduate students.

Is the work clearly and accurately presented and does it cite the current literature?

Yes

If applicable, is the statistical analysis and its interpretation appropriate?

Yes

Are all the source data underlying the results available to ensure full reproducibility?

Yes

Is the study design appropriate and is the work technically sound?

Yes

Are the conclusions drawn adequately supported by the results?

Yes

Are sufficient details of methods and analysis provided to allow replication by others?

Yes

Reviewer Expertise:

Pedagogy, quality of education, evaluation and curriculum design, development of teaching skills, coordination of academic leaders. Currently the general director of the Institute for Educational Evaluation and Development; and President of the Board of Directors of CLUB UNESCO COMPSE.

I confirm that I have read this submission and believe that I have an appropriate level of expertise to confirm that it is of an acceptable scientific standard.

# Peer review: Reviewer response for version 1

**DOI:** 10.5256/f1000research.58874.r100442

**Published:** 2021-12-22

**Authors:** Betsi Josefina Fernández

**Affiliations:** 1 Universidad Pedagógica Libertador, Caracas, Venezuela

It is suggested that the following aspects be reviewed and clarified:

 (a) It is necessary to provide more details of the method applied, differentiating the technique (survey) used according to the instrument designed (questionnaire) and the analysis carried out to facilitate its replicability.

 Among the details related to the method applied, it should be indicated which research paradigm was used, which approach predominated in the methodological approach, and which phases or stages were developed during the fieldwork, which will allow a broad assessment and understanding of the work carried out, since the method indicates the path followed to arrive at the results or findings and its description facilitates the understanding and contextualization of the problem addressed.

 With the complete visualization of the research, being that the technique reflects the procedure in the collection of data and the instrument the one with which the data were collected, and how its validity and reliability of said instrument was carried out.

 b) The statistical analysis (very well developed) is adequate, but not sufficient; it is suggested to refine its interpretation: describe, infer and conclude in each of the tables. However, all the data in the article were tested in the Zenodo open access repository, where it is described ^1^.

 Statistical analysis is the key to visualize the relevance of the results, so it is recommended to describe each table, infer from the data shown and close with a conclusion from each table to then generalize.

 (c) The conclusions drawn are adequately supported by the results, but can be further strengthened if they are confronted with the theoretical elements developed with the objectives of the study, i.e., produce a discussion of the results confronting them with the proposed objectives.

Is the work clearly and accurately presented and does it cite the current literature?

Yes

If applicable, is the statistical analysis and its interpretation appropriate?

Yes

Are all the source data underlying the results available to ensure full reproducibility?

Yes

Is the study design appropriate and is the work technically sound?

Yes

Are the conclusions drawn adequately supported by the results?

Partly

Are sufficient details of methods and analysis provided to allow replication by others?

Partly

Reviewer Expertise:

Pedagogy, Education Curriculum

I confirm that I have read this submission and believe that I have an appropriate level of expertise to confirm that it is of an acceptable scientific standard.

## References

[1] : Pedagogy of success: Perception in undergraduate and postgraduate students at a Peruvian university [Data set]. *Zenodo* .2021; 10.5281/zenodo.5525136 10.5281/zenodo.5525136 PMC872902435035897
